# Understanding the level of animal welfare and associated factors among cat owners in Banyuwangi, Indonesia

**DOI:** 10.14202/vetworld.2024.1210-1215

**Published:** 2024-06-08

**Authors:** Cynthia Retno Wulandari, Azhar Burhanuddin, Putri Laura Faradina, Prima Ayu Wibawati, Abzal Abdramanov

**Affiliations:** 1 Department of Health and Life Sciences, Division of Veterinary Public Health, Faculty of Health, Medicine, and Life Sciences (FIKKIA), Universitas Airlangga, Surabaya, Indonesia; 2 Department of Veterinary Sanitary Expertise and Hygiene, Faculty of Veterinary Medicine, Kazakh National Agrarian Research University, Almaty, Kazakhstan

**Keywords:** animal welfare, cat, education, owner, understanding

## Abstract

**Background and Aim:**

The “Five Freedoms” concept has shaped the development and implementation of animal welfare assessments. This study aimed to analyze the determinants that impact the understanding of animal welfare among individuals who own cats in Banyuwangi, East Java, Indonesia.

**Materials and Methods:**

Questionnaires and interviews were used in this study to gather data from cat owners. One hundred cat owners in Banyuwangi Regency took part in the study. The classification considered factors such as gender, age, education level, occupation, income, and number of cats. The questionnaire passed both validity and reliability tests and was thus deemed suitable for data measurement. The Chi-square test was employed to investigate relationships within the dataset.

**Results:**

A statistically significant correlation (p < 0.05) was established between cat owners’ level of education, occupation, monthly household income, and their grasp of animal welfare, with the number of cats in their household being a determinant factor. Some cat owners in Banyuwangi undervalue veterinarians’ role in treating and preventing feline health issues. The cat owners’ understanding of animal welfare was unaffected by their age or gender. 59% of the cat owners had a low understanding level, 22% had a moderate understanding level, and 19% had a high understanding level.

**Conclusion:**

In Banyuwangi Regency, 59% of cat owners lack understanding of animal welfare concerns. An owner’s educational background, job, income, and whether they own a cat impact their comprehension of animal welfare principles. Limited awareness of animal welfare and veterinarians’ roles exists in Indonesia. It is crucial to educate government officials, veterinarians, and regional leaders about animal welfare for the sake of sustainability. A complete understanding of this topic demands rigorous research, comprehensive studies, and reproducibility. Equally important is effective publicity for the cat population.

## Introduction

An animal is considered healthy, comfortable, well-fed, safe, free to behave naturally, and without fear, pain, or distress, indicating a favorable state of welfare. The determination of animal welfare hinges on their natural behavior [1, 2]. An animal’s welfare is assessed by examining its current quality of life. The animal welfare principle determines proper human conduct toward animals, as per outlined rules [3]. Animals under human care should be granted the five freedoms, ensuring they are free from hunger, thirst, fear, distress, physical discomfort, pain, injury, and disease and able to exhibit normal behaviors. The “Five Freedoms” approach is a result-based method for assessing and enhancing animal welfare [4].

Animals whose lives are impacted by human activities and whose welfare is essential are to be prioritized, considering their well-being and positive emotions closely linked [5]. Our understanding of the psychological processes contributing to favorable human views on animal welfare is still incomplete. The implementation of animal welfare principles should be realized in all groups of animals, both experimental animals, farm animals, and wild animals protected by conservation organizations, to those that are most in demand by the whole community, namely companion animals or pet animals [2]. The types of pet animals in Indonesia are very diverse; however, the most popular pets in Indonesia are cats (47%), followed by fish (22%) birds (18%), and then dogs (10%); furthermore, in 2021, there will be an increase in ownership of pets in Indonesia (38%) compared to 2018 [6].

The application of animal welfare principles to cats in Indonesia, home to a vast population, is essential due to numerous welfare factors. Maintaining a strong human-animal bond and maximizing cat welfare necessitate the owner’s positive attitude and extensive knowledge [7]. A cat owner’s understanding and experience with animal welfare shape their approach to raising their cats. Animal welfare requires a blend of theoretical knowledge and hands-on experience. The study arose due to veterinarians’ worry over new animal welfare concerns. Thoroughly assessing cat owners’ understanding of animal welfare is vital for boosting community engagement in its enforcement.

This study aimed to analyze the determinants that impact the understanding of animal welfare among individuals who own cats in Banyuwangi, East Java, Indonesia.

## Materials and Methods

### Ethical approval and Informed consent

This research proposal was approved by the Universitas Airlangga Faculty of Public Health’s Human Research Ethics Committee under number 73/EA/KEPK/2022. Verbal informed consent was obtained from each participant before the study.

### Study period and location

The study was conducted from March to December 2022. In Banyuwangi Regency, Indonesian residents with Indonesian identity cards were the survey and interview subjects.

### Respondents

One hundred respondents participated in this research. The sample size was determined by the Lemeshow formula and consisted of honest and co-operative Banyuwangi Regency cat owners who owned and cared for cats.

### Questionnaire development and content

The questionnaire for assessing one’s knowledge about animal welfare contained 50 closed-response questions, focusing on the basic comprehension of the “Five Freedoms.” Questions address essential aspects such as housing, nutrition, health care, disease prevention and treatment, comfort, and pain relief. The questions were derived from previously published questionnaires [8, 9] to test and challenge the original hypotheses.

The questionnaire’s essential components were clarified in the explanation section.

1. Demographic information such as gender, age, address, education level, occupation, monthly income, and number of cats owned were collected during the study.
2. Freedom from hunger, malnutrition, and thirst: information regarding pet cats’ food and drink: Type of food, frequency of feeding and drinking, feeding station amount of food and drink, food storage, availability and source of drinking water, owner’s actions regarding remaining cat food and drink [8].
3. Providing adequate space, facilities, bedding, sand, food, water, play areas, and opportunities for outdoor play prevents heat stress and discomfort in cats.
4. Providing adequate space, facilities, bedding, sand, food, water, play areas, and opportunities for outdoor play prevents heat stress and discomfort in cats.
5. Owners should ensure regular veterinary care, proper isolation during illness, vaccination and worming schedules, vitamin supplements, and regular grooming, including bathing, mouth and teeth cleaning, ear cleaning, and nail trimming for optimal cat health. Regularly cleaning the litter box, especially during a cat’s illness.
6. Providing the cat with various opportunities, such as outdoor time, interactions with owners, and solo or social play. Providing periodic mating opportunities, interactive toys, and scratching spots satisfies cats’ behavioral needs.

### Questionnaire validity and reliability test

The instrument’s validity and reliability were assessed using the Statistical Package for the Social Sciences (SPSS) 26.0 software (IBM Corp., NY, USA) and Microsoft Excel 2007 (Microsoft Office, Washington, USA). Thirty external respondents were selected for validity tests in this study. The Pearson test was used for validity analysis in this study. Furthermore, in reliability testing, all items in the question sheet have high reliability (Cronbach’s Alpha value above 0.70), so the questionnaire used has stable consistency (reliability) to be used as a measuring tool [10].

### Assessment and scoring

Researchers employed the Guttman coding method to evaluate pet cat-related responses from the animal welfare questionnaire [11]. The final score was determined by averaging the percentages from the three categories as shown in Table-1.

**Table-1:** Score understanding of five aspects of animal welfare.

Score (%)	Understanding level	Description
>80	High	Very understand
60–80	Moderate	Understand
<60	Low	Do not understand

Score %=(number of correct answers/number of questions) × 100%

Score = (number of correct answers/number of questions) × 100%

### Statistical analysis

Using Microsoft Excel version 2007+, data from interviews and questionnaires were analyzed using the Chi-square statistical test, interpreting and correlating the findings to the research objectives’ variables. The Chi-square test was conducted using SPSS version 26.0.

## Results

### The level of understanding of cat owners on each aspect of animal welfare (the “Five Freedoms”)

56%, 52%, and 60% of respondents showed a limited understanding of freedom from hunger, thirst, and malnutrition; freedom from physical and thermal discomfort; and freedom from pain, injury, and disease, respectively; while 47% and 74% possessed a moderate understanding of freedom from fear and distress and freedom to express normal patterns of behavior, respectively. Specifics are presented in Table-2.

**Table-2:** The level of understanding of cat owners on each aspect of animal welfare (the “five freedoms”) (n=100).

Category aspects of animal welfare	Understanding level (%)*			Total (%)

	High	Moderate	Low	
Freedom from hunger, thirst, and malnutrition	12	32	56	100
Freedom from fear and distress	28	47	25	100
Freedom from physical and thermal discomfort	20	28	52	100
Freedom from pain, injury, and disease	16	24	60	100
Freedom to express normal patterns of behavior	8	74	18	100

* Value of high= >80, moderate= 60–80, low= <60

### Level of understanding of the animal welfare concept

100 cat owners, categorized as respondents, demonstrated a lack of comprehension regarding their cats’ welfare. Cat owners’ perspectives on their feline’s welfare are presented in Table-3.

**Table-3:** The level of understanding of cat owners regarding the welfare of their cats.

No.	Understanding Level	Respondent	

		n	%
1.	High	19	19
2.	Moderate	22	22
3.	Low	59	59

*Value of high= >80, moderate= 60–80, low= <60

### Risk factors that affect the level of understanding of cat owners regarding animal welfare

Traits outlined in Table-3 influence a respondent’s understanding of animal welfare. With p < 0.05, education, occupation, monthly income, and pet ownership independently affected the degree of comprehension. Neither the owner’s gender nor age significantly affected their comprehension of their cat’s well-being (p = 0.254 for gender, p = 0.444 for age).

## Discussion

The “Five Freedoms” represent fundamental criteria for animal welfare, guaranteeing freedom from hunger, thirst, malnutrition, fear, distress, physical and thermal discomfort, pain, injury, and disease, as well as the opportunity to exhibit normal behaviors. The five freedoms establish the fundamental necessities for animal welfare across physical, mental, and natural realms. 56% of respondents do not grasp the idea of being free from hunger, thirst, and malnutrition. According to O’Halloran *et al*. [12], cat owners should be knowledgeable about the appropriate type and amount of food and water for their cats. The considerations for home-prepared diets and the amount of feed required for each cat are different and are influenced by particular pet life stages, breeds, physiological state, animal activity, and disease conditions [13]. Selecting the appropriate feed is essential for fulfilling nutritional needs related to hunger and dehydration. As they grow up, cats can consume both wet and dry food to aid in their development [14]. It is ideal for cats to consume canned food or a home-prepared balanced diet instead of dry food [15]. During discussions on cats’ various eating habits, cat owners highlighted the importance of quality. Cat owners in Banyuwangi Regency are uncertain about the right portion sizes for their cats according to age and weight despite their familiarity with feeding routines. Determining the right amount of food for cats poses a challenge for their owners. According to Villaverde and Chandler [16], ensuring a pet’s diet matches its requirements can prevent overfeeding and obesity. In the study, cat owners overlooked the hazards of giving leftovers to their felines. Feeding cat leftovers may reduce its appetite. Uneaten canned wet food must be discarded within 4 h and opened wet food must be stored in the refrigerator for 48–72 h so that it does not rot and cause poisoning if consumed by animals [17]. Cat owners usually underestimate the importance of offering multiple water sources for their felines and provide constant access to clean and fresh water for a cat to promote its health. It is essential for the cat to eat its preferred food, more so when it refuses to do so. To boost their cats’ appetites, owners resorted to appetite stimulants, hand-feeding, favored foods, and various other methods.

52% of cat owners showed poor understanding of their cats’ needs for temperature and comfort. Cat owners acknowledge the importance of a cage for their pet but remain uncertain about the optimal bedding type. A cage’s design must effectively retain animals while eliminating dangerous features such as sharp edges and gaps. Loberg and Lundmark [18] found that cage size influences cats’ animal welfare measurements. Cat owners frequently overlook indicators of stress and unease in their felines. Assessing the cat’s environment may be necessary for deciding whether to move or improve ventilation to optimize the cat’s living conditions. Ensuring proper ventilation is crucial for managing air quality, controlling heat and humidity, and limiting ammonia concentrations [19].

More than half of respondents struggled to understand the meaning of “pain, injury, and disease-free.” Neglecting their health is a common mistake among cat owners. Monteiro and Steagall [20] discovered that the complexity of pain results in modifications of an animal’s behavior and overall health. Preventing illness and accurately diagnosing and treating conditions are necessary for being free from pain, injury, or disease. Veterinarians must conduct medical examinations for this condition. In Indonesia, many cat owners overlook the significance of vets in preventing and managing feline diseases. This is in accordance with the results of a survey conducted by Laski *et al*. [21], which stated that pet owners in Indonesia showed a significant tendency to use internet searches as the primary source of high-quality information for pet care, with a substantial proportion, namely 71% (207/292) of respondents. 120 out of 292 respondents (or 41%) chose a veterinarian.

Freedom from fear and distress was understood to the degree of 47%, while 74% was the degree of understanding for freedom to express normal patterns of behavior. A cat’s ears lose their upright position and its body curls when it is afraid [22]. Ellis [23] observed that when cats are frightened, they assume a crouched posture by bending downward and drawing in their bodies toward the ground. The pupils will dilate, accompanied by a growling sound, indicating fear [24]. Cats exhibit fear through sudden jumping, running, and hiding [25].

Research shows cat owners should allow their felines to maintain normal behavioral patterns. Cat owners value having an open space for their feline companions to play, roam, and display their natural behaviors. Cat owners should allow their cats sufficient freedom yet ensure careful supervision, especially outside enclosed spaces. Owners must supervise their pets to prevent the transmission of infectious diseases from other animals [26]. According to Nagasawa *et al*. [27], 5–10 min of daily cat play can help decrease behavior problems. Interacting effectively during playtime enhances a beneficial bond between owners and their animals [28]. Cat owners often overlook the importance of providing interactive toys to their felines. The benefits of cat toys are that they can reduce signs of negative behavior or stress and train cats to be more active to minimize the possibility of obesity [29]. These toys, such as toy mice, ping-pong balls, fishing rods, and others, encourage normal feline behavior. By providing scratching areas, cat owners can cater to their pets’ natural instincts.

Cats commonly damage household items through scratching. The predominant issues of unwanted feline behaviors are house soiling and aggression [30]. The most frequently reported problem behavior by cat owners was scratching furniture (47.5%), followed by aggression during play (40.2%), excessive vocalization (37.6%), aggression toward unfamiliar cats (36.6%), and being overly active at night (35.8%) [31]. Cats typically exhibit such behavior to draw owners’ attention [32]. Cat owners utilize different strategies, including limiting access to potential hazards, hiding targeted objects, and employing positive reinforcement, to prevent damage in their homes by their pets. The use of positive punishment techniques led to more instances of unwanted scratching, while the implementation of enrichment and positive reinforcement methods decreased the occurrence of unwanted scratching [33]. Reported methods for correcting feline misbehavior through punishment were hitting or kicking. Household pet research reveals fear-based aggression and compromised welfare can result from positive punishment [34]. To avoid fear and defensiveness in cats, it is recommended to avoid using physical punishment during research, although studies on this relationship in felines are limited [35, 36].

Animal welfare is a scientifically valid concept. The perception of animal welfare and the scientific methods applied to evaluate and enhance it are shaped by subjective value judgments [37]. Our concept integrates scientific evidence with ethical values. 59% of respondents failed to grasp animal welfare concepts based on the research findings. Failure to care for pets properly can negatively impact their health. The variables of educational level, occupation, monthly income, and number of cats within a household influence cat owners’ perception of animal welfare (Table-4). Those with a diploma or bachelor’s degree had a greater understanding than respondents with a senior high school education or less.

**Table-4:** Characteristics of respondents that influenced the level of understanding of animal welfare (n = 100).

Variable	Number of respondents based on the level of understanding of the animal welfare			Total	p-value

	High	Moderate	Low		
Gender					
Male	9	5	21	35	0.254
Female	10	17	38	65	
Age (years)					
<20	0	0	5	5	0.444
21–50	15	18	44	77	
>50	4	4	10	18	
Level of education					
Not yet/not in school	0	0	2	2	<0.001*
Did not finish elementary school or equivalent	0	0	4	4	
Elementary school or equivalent	0	1	15	16	
Junior high school or equivalent	0	0	13	13	
Senior high school or equivalent	3	12	22	37	
Diploma I/II/III degree	10	6	2	18	
Diploma IV degree	5	3	1	9	
Bachelor’s degree	1	0	0	1	
Occupation					
Unemployed	2	4	22	28	<0.001*
Farmers	0	0	6	6	
Farm or plantation labor	0	0	4	4	
Honorary employee	0	4	1	5	
Private sector employee	3	2	2	7	
State-owned enterprises employee	4	0	0	4	
Entrepreneur	6	11	15	32	
Trader	0	1	9	10	
Civil servants	4	0	0	4	
Monthly income (Rupiah)					
>Rp 1,500,000	0	4	32	36	<0.001*
Rp 1,500,000–2,500,000	1	11	22	34	
Rp 2,500,000–3,500,000	6	6	5	17	
>Rp 3,500,000	12	1	0	13	
Number of cats in the household					
1–5	15	22	58	95	0.008*
6–10	2	0	1	3	
>10	2	0	0	2	

* Indicates a significant difference (p < 0.05)

In the moderate and high understanding categories of cat welfare, a larger proportion of diploma-level or above respondents fell (Table-4). 28% of respondents with a high education level were more inclined to understand animal welfare concepts. Individuals with advanced education levels reported experiencing twice the joy from their pets compared to those with less education. Users with higher formative levels derive more pleasure from engaging with their pets. Education significantly shapes an individual’s knowledge and actions. Cat owners with a low level of education tend not to understand the concept of animal welfare [38]. Education enables the development of social and emotional intelligence, promoting personal growth and self-awareness. Through education, individuals obtain knowledge and undergo personal transformation, leading to societal harmony. A person’s behavior and lifestyle are significantly influenced by their education level. The more educated a person is, the more effectively they process information, resulting in expanded knowledge and comprehension. Education shapes individuals’ knowledge and behavior. Cat owners with less education are more likely to neglect cats’ welfare.

Household income influences individual lifestyles [39]. A person’s income and economic status determine their access to necessary facilities and thus their consumption levels. The Governor of East Java declared a minimum wage of USD 157.54 per month for Banyuwangi Regency in 2023 [40]. Seventy percent of cat owners having an income below the minimum wage may compromise animal welfare due to financial constraints. In multicatted households, unrelated felines separate resources to minimize overlap and potential conflicts, by occupying distinct areas in the house for resting and feeding at different times of the day [41]. Cats with varied household populations exhibited similar rates of reported behavior problems [8].

## Conclusion

In Banyuwangi Regency, 59% of cat owners have a low level of knowledge about animal welfare. Household variables influence owner comprehension, such as education level, occupation, monthly income, and cat ownership. The fundamental effort to increase cat owners’ knowledge and the appropriate intervention is to conduct education about animal welfare. Raising Indonesian public awareness of animal welfare and veterinarians’ role in promoting sustainability is crucial for Indonesia’s sustainability efforts.

## Data Availability

The supplementary data including the questionnaire can be available from the corresponding author.

## Authors’ Contributions

CRW and PAW: Conceptualization and methodology. CRW, AB, and PLF: Interviewed the respondents and collected the data. PLF and AA: Analyzed the data. PAW, AB, and AA: Wrote the manuscript with input from all authors. All authors have read and approved the final version of the manuscript.
